# CDK5: Key Regulator of Apoptosis and Cell Survival

**DOI:** 10.3390/biomedicines7040088

**Published:** 2019-11-06

**Authors:** Rabih Roufayel, Nimer Murshid

**Affiliations:** College of Engineering and Technology, American University of the Middle East, Kuwait

**Keywords:** CDK5, p25 phosphorylation, p35, p39, neural apoptosis

## Abstract

The atypical cyclin-dependent kinase 5 (CDK5) is considered as a neuron-specific kinase that plays important roles in many cellular functions including cell motility and survival. The activation of CDK5 is dependent on interaction with its activator p35, p39, or p25. These activators share a CDK5-binding domain and form a tertiary structure similar to that of cyclins. Upon activation, CDK5/p35 complexes localize primarily in the plasma membrane, cytosol, and perinuclear region. Although other CDKs are activated by cyclins, binding of cyclin D and E showed no effect on CDK5 activation. However, it has been shown that CDK5 can be activated by cyclin I, which results in anti-apoptotic functions due to the increased expression of Bcl-2 family proteins. Treatment with the CDK5 inhibitor roscovitine sensitizes cells to heat-induced apoptosis and its phosphorylation, which results in prevention of the apoptotic protein functions. Here, we highlight the regulatory mechanisms of CDK5 and its roles in cellular processes such as gene regulation, cell survival, and apoptosis.

## 1. Introduction

The proline-directed serine/threonine cyclin-dependent kinase 5 (CDK5) is an atypical member of the well-studied family of cyclin-dependent kinases (CDKs) [1]. CDK5 was first identified by Hellmich and coworkers as a neuronal cdc2-like kinase (nclk) [2] due to its ability to phosphorylate the lysine–serine–proline (KSP) motif of neurofilaments in vitro and shares 58% and 61% amino acid sequence homology to mouse CDK1 and human CDK2 [2]. CDK5 was also reported as tau protein kinase II (TPKII) due to its association with and ability to phosphorylate tau [3,4,5]. It is reported that CDK5 phosphorylates tau at the hyperphosphorylated sites in Alzheimer’s disease (AD) brains [6,7]. Gong and co-workers detected the phosphorylation of tau at each specific site using Western blots with different site-specific and phosphorylation-dependent tau antibodies [8]. They found that CDK5 phosphorylates the AD-tau at Thr-181, Ser-199, Ser-202, Thr-205, and Ser-404 [8].

Lew et al. reported the same kinase as brain proline-directed protein kinase due to its functional similarity to cdc2 in the bovine brain [9]. In 1993, Kobayashi et al. identified that the 30 kDa protein subunit of TPKII was the active enzyme and termed it as CDK5 [10]. CDK5 has been mapped to 7q36 within the human genome. Translation of the 987 bp CDK5 transcript yields a 33 kDa protein that phosphorylates target proteins on serine and threonine residues within a S/TPXK/R motif, where X is any amino acid and P is a required proline residue at position +1 [1,11]. CDK5 appears to have no intrinsic cellular distribution, instead it tends to co-localize with its substrates and activators [12,13,14]. Being a member of the CDK family, CDK5 shares structural features and characteristics with other CDKs, though its activation pattern is strikingly different [15,16].

## 2. Activators of CDK5

**Figure 1:**
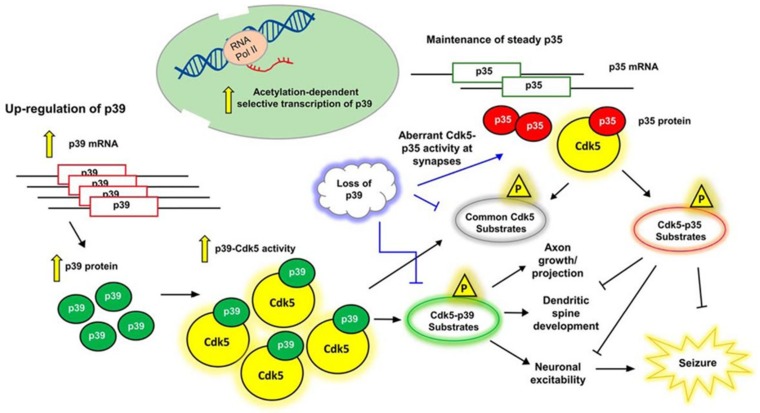
Functional diversity and cooperation by Cdk5 (cyclin-dependent kinase 5) activators in postnatal brain neurons (Adopted from reference 29) [29].

Unlike other CDKs that require the binding of cyclins in order for their activation, CDK5 requires the binding of p35, p39, or p25 (a proteolytic fragment of p35) for activation. p35 (NCK5a, neuronal CDK5 activator) was first discovered due to its association and activation of CDK5 [17,18,19]. However, p39 (NCK5ai, neuronal CDK5 activator isoform) was first identified as a 39 kDa isoform of p35 that shared 57% amino acid homology with p35 [20], p25 was first discovered as a truncated form of p35 that was found in the neurons of Alzheimer patients [21], and subsequent studies identified that cleavage of p35 into p25 was calpain- and dephosphorylation-dependent [22,23,24]. Lastly, p29, a similarly cleaved product of p39, has also been identified and is known to play a role in the deregulation of CDK5 [25]. p35, p39, and p25 show limited amino acid sequence homology to cell-cycle cyclins, though they are able to interact with CDK5 by folding into a tertiary structure containing a CDK5-binding domain that is similar to the CDK-binding domains of other cyclins [15,16,26,27,28]. Studies regarding the age and regional distribution of p35 and p39 in embryonic and postnatal rat brains have demonstrated that the expression pattern of p35 and CDK5 is the inverse of p39, suggesting that they might have a developmental stage- and region-specific function [29,30]. The functional diversity and cooperation by Cdk5 activators in postnatal brain neurons has been discussed by Wenqi and coworkers [29]. As shown in Figure 1, p39 transcription is enhanced by histone acetylation in brain neurons, leading to the upregulation of both p39 mRNA and protein levels, whereas p35 abundance is unaltered. Phosphorylation of CDK5 substrates by p39-dependent Cdk5 governs axonal and dendritic spine neuronal development, thereby inhibiting p35–Cdk5 complex roles. Neural development abnormalities result due to the loss of p39, leading to seizures and sporadic adult lethality in p35^−/−^ mice, however seizures are reduced upon p39 activation in p39^−/−^ mice with no detectable abnormalities.

**Figure 2:**
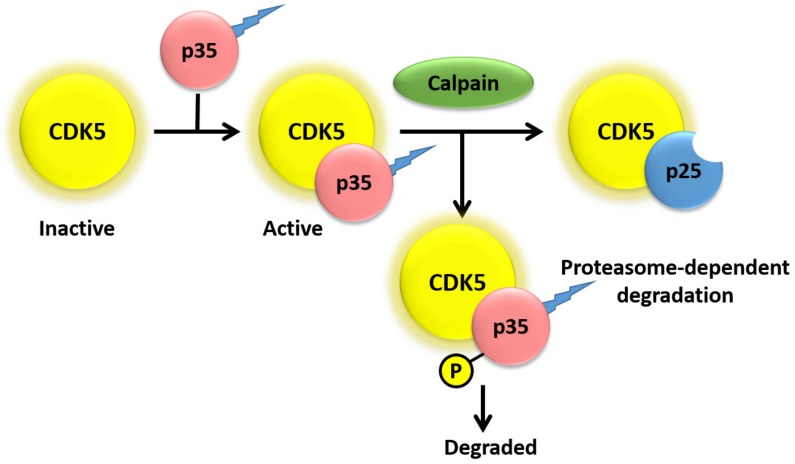
CDK5 activation is initiated by binding to the myristoylated regulatory subunits.

Interestingly, the subcellular localization of CDK5 is dependent on the activator to which it is bound. Specifically, CDK5 remains primarily cytosolic when it is not bound to any activators, whereas interaction with either p35 or p39 results in localization of CDK5, primarily to the plasma membrane and perinuclear region, due to myristoylation of p35 and p39, though CDK5/p35 complexes can also be seen in the cytosol [31]. As shown in Figure 2, CDK5/p35 complex is localized to the plasma membrane and cytosol, limiting the action of complex to specific cellular compartments for degradation. Calpain cleavage of p35 increases the production of CDK5/p35 complex that is resistant to degradation, therefore allowing its interaction with different targets. It has been observed that p39 can mask p35 deficiency by compensating of some, but not all, functions of p35 in p35^−/−^ mice [13,32]. On the other hand, p39^−/−^ mice show no detectable abnormalities [12]. However, p35/39^−/−^ double-knockout mice show a more severely affected phenotype, as compared with p35^−/−^ mice, which is identical to CDK5^−/−^ mice [12]. Due to lack of CDK5 activity and the severe phenotypic abnormalities in p35/39^−/−^ mice, p35 and p39 are thought to be necessary and sufficient for both normal CDK5 activity and proper neurodevelopment [1,12,33]. Studies have also found that cyclin D and E are able to bind to CDK5, though these associations do not activate CDK5 kinase activity [34,35,36] Lastly, CDK5 has been reported to bind and be activated by cyclin I, which is predominantly expressed in terminally differentiated cells, such as podocytes and neurons [37]. Binding of cyclin I to CDK5 has been proposed to have an anti-apoptotic function that is specific to terminally differentiated cells, possibly due to activation of the MEK/ERK pathway and increased expression of Bcl-2 and Bcl-xL [37,38].

## 3. Regulation of CDK5 Activity

The kinase activity of CDK5 is primarily regulated by the presence, amount, and balance among available p25, p35, and p39 [14], p35 and p39 levels are controlled by the balance between synthesis and degradation, whereas p25 levels are direct results of calpain-dependent cleavage of p35 [22,24,39,40]. Treatment of cultured rat embryonic brain neurons, and medium-sized spiny neurons, with brain-derived neurotropic factor (BDNF) results in ERK-mediated expression of p35 [41,42].

Treatment of PC12 cells (rat pheochromocytoma) with nerve growth factor (NGF) results in the strong induction of p35 expression that is ERK-dependent and mediated by early growth response protein 1 (Egr1) [43]. In support, Egr1^−/−^ mice reduce p35/p25 protein levels and CDK5 activity in brain tissues [44]. Chang et al. identified that both p35 and p39 are targets for the transcription factor, heat shock factor protein-2 (HSF2), by observing reduced p35/p39 protein levels in the telencephalon of Hsf2^−/−^ mice during cortical development [45]; p35 and p39 have overlapping, but distinctive, expression profiles in the developing mouse brain. Expression of p35 can be observed in the cerebral cortex as early as embryonic day 15, whereas p39 expression is not present until postpartum day 0 (P0). Furthermore, expression of p35 decreases while expression of p39 is increased after P0 in the cerebral cortex, while expression of both p35 and p39 is highest during embryonic development and decreases after birth in the cerebellum, brain stem, and spinal cord [46]. In the adult rat, expression of p35 mRNA is predominantly greater in the brain as compared with the spinal cord, whereas expression of p39 mRNA is the opposite of p35 [47].

Protein levels of p35 and p39 are also regulated by protein degradation. Studies have shown that p35 has a half-life of ~30 min and is ubiquitinated in COS-7 cells (monkey kidney fibroblast), mouse primary cortical neurons, intracerebral hemorrhage (ICH) models of rats, and primary cortical neurons [40,48,49]. Furthermore, phosphorylation of p35 at Thr138 by activated CDK5 results in proteasome-dependent degradation of p35 (Figure 2), resulting in a negative feedback loop that inhibits CDK5 activity [24,36,48]. Conversely, phosphorylation of p35 residues Ser59, Ser65, and Ser124 by protein kinase Cδ results in stabilization of p35 [50]. A recent study also showed CDK5 increases MARK4 activity through tau phosphorylation at Ser262 [51]. Lastly, prevention of protein phosphatase 1 and 2A activity by treatment of COS-7 cells with okadaic acid results in maintained phosphorylation of Thr138 and prevention of calpain-dependent cleavage of p35 into p25 [24]. One study investigated the relative rates of degradation of p35 as compared with p39 and observed that p39 both cleaved and degraded at a slower rate than p35 [52]. The variance in degradation and cleavage rates were attributed to the N-terminal p10 regions of p35 and p39 that, as a result of membrane association predominantly controlled by myristoylation, regulate the cleavage and degradation of p35 and p39 respectively [52]. Lastly, the cleaved products of p35/p39, p25/p29 respectively, are protected from degradation as they lack the N-terminal p10 region. This leads to aberrant subcellular localization and interaction of p25/p29 with CDK5, which results in deregulation of CDK5 target specificity and promotes neurodegeneration [23,53,54].

In addition to levels of p25/p35/p39 protein, CDK5 activity is also regulated by direct phosphorylation of CDK5, though the effect of CDK5 phosphorylation on CDK5 activity is dramatically different from that of other CDKs. Commonly, CDK activity is inhibited by phosphorylation of both Thr14 and Tyr15 by the dual specific kinases Wee1 and myelin transcription factor 1, while phosphorylation of CDK1/2 on residue Thr160/161 by CDK-activating kinase (CAK) is required for activation of CDK1/2 activity [33,36,53]. Conversely, CDK5 does not require phosphorylation of Ser159 (equivalent to Thr160/161 in CDK1/2), as CDK5, prepared from E. coli, becomes activated upon binding with p35, and the addition of CAK does not result in phosphorylation or enhanced kinase activity of CDK5 [55,56]. Analysis of the crystal structure of CDK5/p25 complexes demonstrates that binding of p25 forces the activation loop of CDK5 to adopt an open conformation that is typical of other activated proline-directed kinases, such as CDK1/2, once phosphorylated [16,33,36]. In addition, CDK5 is not phosphorylated at Tyr15 by Wee1, although phosphorylation of Thr14 by CDK T14 kinase, purified from bovine thymus, results in inactivation of CDK5 [56,57]. In opposition to the inhibitory role of Tyr15 phosphorylation for CDK1/2, phosphorylation of Tyr15 results in increased CDK5 enzymatic activity and is mediated by non-receptor Src family tyrosine kinases, such as c-Abelson (c-Abl) and Fyn, and the receptor-type tyrosine kinases ephrin type-A receptor 2/4 [58,59,60]. Phosphorylation of Tyr15 by c-Abl is mediated by CDK5 and Abl enzyme substrate (Cables), which facilitate interaction between CDK5 and c-Abl and lead to enhanced Tyr15 phosphorylation of CDK5 [58]. Phosphorylation of Tyr15 by Fyn is mediated by association of Plex-A2 with both active Fyn and CDK5, resulting in CDK5 phosphorylation and increased kinase activity that is dependent upon Fyn activity [59]. Molecular dynamic simulations have predicted that phosphorylation of Tyr15 allows for ATP to adopt a more favorable conformation within CDK5, allowing for enhanced substrate phosphorylation. It is currently unknown whether phosphorylation of Tyr15 plays a direct role in the binding of CDK5/p35, therefore having control over CDK5 activity, or if Tyr15 phosphorylation only affects substrate specificity and/or the rate of kinase activity [33].

CDK5 activity can also be inhibited by various pharmacological compounds. The earliest, and currently the most popular, inhibitors of CDK5 are the purine-based compounds olomoucine and roscovitine [61,62,63]. Though, due to the interaction of these purine-based compounds with the conserved ATP-binding pocket present in all members of the CDK family, both olomoucine and roscovitine are not selective inhibitors of CDK5 [53,64]. Various targets for both compounds have been identified, where olomoucine and roscovitine have been shown to inhibit CDK1, CDK2, CDK5, Erk1, and Erk2, while in addition, roscovitine is able to also inhibit pyridoxal kinase [61,62,65,66,67]. These compounds are still cited as “specific” inhibitors of CDK5, though information regarding the semispecific binding properties of olomoucine and roscovitine has been previously published, and subsequently, companies, such as Boehringer Ingelheim and Pfizer, are currently developing more specific CDK5 inhibitors [53].

## 4. Function of CDK5 in Neuronal Development

CDK5 is an essential player in central nervous system development, function, and disease. Its function is indispensable for proper neuronal migration and differentiation, axonal elongation, and synaptic function. Also, association of CDK5 with p25 leads to altered regulation of CDK5 that promotes neuronal apoptosis and development of neurological diseases such as Alzheimer’s and Parkinson’s disease [1,23,36,40,53,68,69]. Studies of CDK5^−/−^ and p35/p39^−/−^ mice have revealed that these mice die during the perinatal period of development due to widespread disruption of neuronal migration in the cerebral cortex, hippocampus, and cerebellum, resulting in a lack of cortical laminar structure and cerebellar foliation [12,53,68,70,71]. The requirement for CDK5 in development and survival of p35-expressing neurons was demonstrated by rescue of neuronal development by re-expression of CDK5, under the control of the p35 promoter, in CDK5^−/−^ mice [72]. Studies investigating the molecular basis for the pathological effect of CDK5 knockout have found that caspase-3 activity is increased in the brain cortex of CDK5^−/−^ mice as compared with wild-type mice [73]. In addition to CDK5^−/−^ mice, p35^−/−^ mice have severe defects in cortical lamination, due to lack of CDK5/p35 kinase activity and improper neuronal migration, and suffer from seizures and sporadic adult lethality [68,69,74]. Though CDK5^−/−^ and p35^−/−^ mice do share similar phenotypic abnormalities, it should be noted that these two phenotypes are not identical as p39 can maintain limited function of CDK5 in p35^−/−^ mice [13,32].

**Figure 3:**
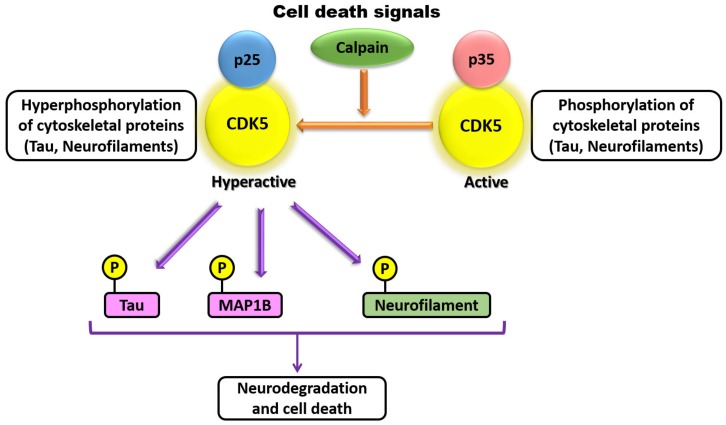
Cyclin-dependent kinase-5 (Cdk5) substrates in brains with neurodegenerative diseases. In a neurodegenerative brain disease, there is Caplain cleavage in the N-terminal region of p35 to a more stable isoform p25, forming a CDK5/p25. This stable complex strongly phosphorylates neurofilaments, Tau, and another microtubule-associated protein, MAP1B compared with CDK5/p35 complex.

CDK5 has also been demonstrated to be involved in axonal elongation. Studies have demonstrated that there exists a high degree of temporal correlation among CDK5 activation, p35 expression, and formation of axonal tracts in the developing brain [1,68,75]. CDK5 and p35 are both present at the leading edge of axonal growth cones in developing neurons, where CDK5 is co-distributed with actin filaments but not with microtubules [76]. siRNA-mediated knockdown of p35 in cultured neurons results in decreased laminin response and inhibition of axonal elongation, which can be rescued by co-expression of p35 but not CDK5 [75,76]. At the molecular level, inhibition or silencing of CDK5 in PC12 cells suppresses phosphorylation of protein phosphatase 1 (PP1) and prevents NGF-induced neurite outgrowth, whereas overexpression of wild-type PP1 promotes NGF-induced differentiation of PC12 cells [44]. Additional studies have also shown that CDK5 may play a role in neurite branching, which is vital for proper neuronal patterning [36,77,78]. Pathological studies revealed that p35 is cleaved in its N-terminal region into p25 by Calpain in Alzheimer’s, Huntington’s, and other degenerative diseases (Figure 3) [22,39,40,79,80].

The proteolytic p25 fragment of p35 exhibits a different subcellular localization with and is more stable that its precursor p35. CDK5/p25 have shown to phosphorylate both Tau and MAP1B found in neurofibrillary tangles (NFTs) compared with CDK5/p35 [19,31]. CDK5 has been shown to have various roles in synapse formation, maintenance, and synaptic communication. A role for CDK5 in the formation of dendritic spines was identified due to inhibition of BDNF-induced dendritic growth when rat primary hippocampal neurons lack CDK5 activity [81,82]. Conversely, CDK5 regulates retraction of dendritic spines by phosphorylating ephexin1 upon being itself phosphorylated on Tyr15 by activated EphA4, leading to activation of the small Rho GTPase RhoA and retraction of dendritic spines [60]. CDK5 has also been identified to regulate neuron secretion at the synapse by phosphorylating key mediators, such as Synaspsin1, Munc18, and Amphipysin, resulting in functional changes in the activity of these key mediators [36,68]. CDK5 and p35 are abundant in embryonic muscle and at the neuromuscular junctions in adulthood [82,83], and additional studies have shown that CDK5, p35, and p39 are present in synaptic membranes [83,84,85]. CDK5 is able to phosphorylate the membrane receptor ErbB, resulting in ErbB endocytosis and increased acetylcholine rector transcription in nearby synaptic sites [83]. CDK5 has also been demonstrated to play a role in the activity of dopamine- and cAMP-regulated neuronal phosphoprotein 32, which controls dopamine signaling in specific neurons in adult mice [68,86]. Lastly, in addition to the roles for CDK5 previously discussed, CDK5 function has also been implicated in many other cellular processes such as cell cycle control and gene regulation, cell survival and apoptosis, membrane dynamics, focal adhesion formation, intracellular trafficking, and glucose metabolism, overall demonstrating the varied and essential roles of CDK5 in proper cell function [1,33,36,68,69,87,88].

## 5. Function of CDK5 in Non-Neuronal Cells

CDK5 has also been identified to play important roles in processes such as cell death and proliferation, angiogenesis, migration of epithelial and cancer cells, inflammation, myogenesis, glucose metabolism, and insulin secretion in non-neuronal cells [53,87,89,90,91,92]. These studies have investigated CDK5 function in cells such as cells of hematopoietic lineage, HEK293, COS7, MEF, HCT116, HeLa, adipoctyes, pancreatic β cells, and many other non-neuronal cell types [53,91]. CDK5 has been demonstrated to be present in both podocytes and pancreatic β cells [38,93,94]. Additionally, both p35 and p39 have been found to be expressed in pancreatic β cells [93,94]. Ubeda et al. observed that elevated extracellular glucose concentration results in increased expression of p35 and a correlative increase in CDK5 kinase activity [93]. CDK5/p35 is able to stimulate the insulin promoter in response to elevated glucose levels as inhibition of CDK5 prevents stimulation of the insulin promoter [89,93]. Additionally, suppression of CDK5 and p39 results in inhibition of Ca^2+^-induced insulin exocytosis in pancreatic β cells [94].

The function of CDK5 has also been studied in cells of hematopoietic lineage and has been implicated in processes such as response to inflammation, proliferation, and apoptosis [53,95]. Overexpression of a dominant-negative CDK5 in IPC-81 rat leukemia cells results in inhibition of cAMP-induced caspase-3 activation and apoptosis [96]. Additionally, treatment of IPC-81 cells with cAMP results in moderately increased expression of CDK5 that correlates with cAMP-induced apoptosis [96]. Treatment of various B-CLL cell lines (B cell chronic lymphocytic leukemia), which are deficient in p53-dependent apoptosis, with roscovitine results in p53-independent apoptosis within 24 h of treatment due to decreased expression of genes involved in transcription, translation, survival, and DNA repair [97]. One anti-apoptotic protein that has been identified to be down-regulated in roscovitine-treated multiple myeloma (MM) cells is Mcl-1, whereby rapid down-regulation of Mcl-1 transcription and translation is independent of caspase cleavage [98]. The mechanism for Mcl-1 transcriptional down-regulation was later shown to be caused by roscovitine-induced dephosphorylation of RNA polymerase II [99]. Phosphorylation of RNA polymerase II is required for its function, and subsequently, roscovitine-induced dephosphorylation of RNA polymerase II results in inhibited transcription, leading to decreased Mcl-1 levels that were shown to be sufficient for induction of apoptosis in MM cells [99]. In addition to down-regulation of Mcl-1 mRNA, it has been shown that treatment of B-CLL cells with roscovitine induces rapid proteasomal degradation of Mcl-1 and apoptosis [100]. In addition, Mcl-1 remains associated with Noxa when B-CLL cells are treated with roscovitine, and RNAi-induced reduction of Noxa protein levels confers resistance to roscovitine-induced apoptosis in B-CLL cells [100]. Though Noxa has been shown to be phosphorylated by CDK5, it has yet to be determined if roscovitine-induced apoptosis is due to inhibition of Noxa phosphorylation via inhibition of CDK5 [101]. Our studies provided evidence that this atypical cyclin-dependent kinase regulates the expression of miR-23a. Hyperthermic treatment led to a loss of CDK5 tyrosine-15 phosphorylation, which was prevented by HSP70 overexpression. Treatment with the CDK5 inhibitor roscovitine caused an increase in Noxa protein levels and sensitized cells to heat-induced apoptosis [64,102].

**Figure 4:**
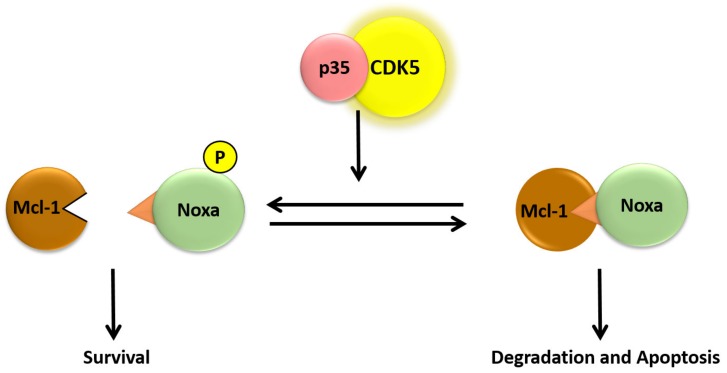
Hypothetical model for the role of Noxa serine-13 phosphorylation on induced apoptosis.

As future research, more studies should be done on the role of CDK5 in protein regulation, as a study has shown that pro-apoptotic BH3-only protein Noxa is required for hyperthermia-induced Mcl-1 degradation and apoptosis [103]. Also, it has been demonstrated that Noxa is phosphorylated on both serine-13 and tyrosin-15 by the CDK5, resulting in prevention of the apoptotic function of Noxa in tumor cells. These findings support that exposure to stress results in decreased Noxa phosphorylation, due to reduced CDK5 activity, leading to activation of Noxa, Mcl-1 degradation, and apoptosis (Figure 4) [101,102]. Therefore, more follow-up studies should focus on the effect of stress on CDK5 phosphorylation and solubility and determine whether Noxa or any other apoptotic protein is phosphorylated on serine-13 in lymphoid cells. CDK5 knockdown or inhibition has been documented to be an anticancer agent since and is generally dysregulated in various cancer cells [92].

## 6. Function of CDK5 in Autophagy

Autophagy is an essential natural regulating mechanism that governs the removal of unnecessary and long-lived cellular components. In healthy mammalian cells, the dominant activity is directed towards intracellular homeostatic turnover of proteins, mediating self-renewal energy generation, metabolism, and cell death [104,105,106]. New evidence has reported that many diseases are connected to autophagy, including primary cancer origins [107]. Autophagy activation insures and protects cancer cells, and this process in known as adaptive autophagy, which is directly associated with CDK5. Cancer cells subjected to chemotherapy treatments have the ability to induce autophagic cell death through the inhibition of Akt [108]. Recent studies have showed that autophagy could be regulated using anticancer drugs. A tumor suppresser protein CDK5RAP3, which is a regulatory subunit of CDK5, has participated in the regulation of human renal cancer. Overexpression of this protein showed a significant activation of autophagy [109]. CDK5 is considered to be an autophagy-regulating kinase due to its enriched overexpression in the central nervous system. Deregulated CDK5 is associated with neuronal death due to endophilin B1 (EndoB1), a CDK5 substrate. Cdk5-mediated phosphorylation of EndoB1 insured neural loss due to autophagy induction in models of Parkinson’s disease [110]. Reduced basal autophagy was observed in an endogenous mutated Acn protein lacking CDK5 function. In contrast, CDK5 activity was elevated and maintained neural health in Acn-prosphyrylated serine 437 in flies, indicating that Acn is a an important substrate of CDK5 for activating autophagy [111]. These findings suggests that CDK5 is important to maintain basal autophagy.

## 7. Conclusions

Regulation of apoptosis is critical for cell survival during stress and for proper removal of aged and damaged cells. Deregulation of apoptosis has been found to be essential for many diseases, including cancer and many neurodegenerative diseases. Post-translational modifications, such as CKD5 phosphorylation, have been shown to regulate the function of various regulatory proteins, including members of the Bcl-2 family of pro- and anti-apoptotic proteins. Hyperthermia is one type of stress that is able to induce apoptosis through activation of the intrinsic pathway and results in changes in phosphorylation and activity of members of the Bcl-2 family. This review highlighted the regulatory mechanisms of CDK5 and its roles in cellular processes such as gene regulation, cell survival, and apoptosis. However, we seek to highlight the mechanisms of this atypical cyclin member phosphorylation that may aid in the hunt for additional targets with potential therapeutic relevance.
